# Novel concepts and engineering strategies for heterologous expression of efficient hydrogenases in photosynthetic microorganisms

**DOI:** 10.3389/fmicb.2023.1179607

**Published:** 2023-07-12

**Authors:** Conrad Schumann, Jorge Fernández Méndez, Gustav Berggren, Peter Lindblad

**Affiliations:** ^1^Molecular Biomimetics, Department of Chemistry - Ångström, Uppsala University, Uppsala, Sweden; ^2^Microbial Chemistry, Department of Chemistry - Ångström, Uppsala University, Uppsala, Sweden

**Keywords:** photobiological hydrogen, [FeFe] hydrogenases, [NiFe] hydrogenases, cyanobacteria, green algae, heterologous expression, genetic engineering, photosynthesis

## Abstract

Hydrogen is considered one of the key enablers of the transition towards a sustainable and net-zero carbon economy. When produced from renewable sources, hydrogen can be used as a clean and carbon-free energy carrier, as well as improve the sustainability of a wide range of industrial processes. Photobiological hydrogen production is considered one of the most promising technologies, avoiding the need for renewable electricity and rare earth metal elements, the demands for which are greatly increasing due to the current simultaneous electrification and decarbonization goals. Photobiological hydrogen production employs photosynthetic microorganisms to harvest solar energy and split water into molecular oxygen and hydrogen gas, unlocking the long-pursued target of solar energy storage. However, photobiological hydrogen production has to-date been constrained by several limitations. This review aims to discuss the current state-of-the art regarding hydrogenase-driven photobiological hydrogen production. Emphasis is placed on engineering strategies for the expression of improved, non-native, hydrogenases or photosynthesis re-engineering, as well as their combination as one of the most promising pathways to develop viable large-scale hydrogen green cell factories. Herein we provide an overview of the current knowledge and technological gaps curbing the development of photobiological hydrogenase-driven hydrogen production, as well as summarizing the recent advances and future prospects regarding the expression of non-native hydrogenases in cyanobacteria and green algae with an emphasis on [FeFe] hydrogenases.

## 1. Introduction

Sunlight is the most abundant and widespread energy source around the globe. Every year the sun irradiates Earth’s surface with more than 7,000 times the current global energy consumption. On average 173 PW of energy strikes Earth’s surface at any time, providing a virtually unlimited energy source that can be harvested locally. The conversion of solar energy into chemical energy, e.g., in the form of hydrogen (H_2_), greatly facilitates storage, distribution and applicability in a wide-range of settings. Presently H_2_ is mainly produced via steam reforming (yielding so-called “grey” hydrogen), but truly fossil-free hydrogen production is increasing. Current fossil-free processes rely on electrolyzers driven by carbon-free electricity. Albeit efficient these technologies feature several limitations, e.g., high demand of electricity and scarce rare earth metals requirements. Renewable energies (which can yield green and yellow hydrogen) have become cost-competitive and their expected growth trends are astonishing [estimated annual growth rates of 8.4% for renewables (Novus-Light-Technologies, 2023)] during the next decade. Similarly, nuclear power (pink hydrogen) is expected to increase by 5.7% (Bussiness-Research-Company, 2023) in the same time period. Still, electricity demands are expected to increase significantly, up to 50%, during the electrification process towards a net-zero carbon emissions economy (IEA, 2021; Exxon-Mobil, 2022). It follows that electrochemical hydrogen production is likely to be limited to contexts where there is a frequent surplus of clean electricity generation. Consequently, novel hydrogen production technologies are required for global large-scale production, ideally integrated within other industrially relevant processes.

Biotechnological hydrogen gas production provides an inexpensive and scalable alternative technology for H_2_ production. Indeed, our drive towards a hydrogen society is pre-dated by evolution, which has developed an efficient hydrogen economy involving biological machineries capable of hydrogen production without the requirement of rare-earth metals and avoiding heavy electricity consumption. Waste-to-hydrogen has been intensively explored as a sustainable alternative to conventional hydrogen production from fossil fuels. However, the demand of high-cost pre-treatment methods, the use of inconsistent feedstock as well as low energy conversion efficiencies are noteworthy constrictions of this technology (Lui et al., 2020). Also, the competition with well-established biogas production processes and the overall reduced energy content in waste limits its application for large-scale H2 production. Instead, oxygenic photosynthetic organisms stand out as ideal platforms for biological H_2_ production as they are capable of direct solar-to-hydrogen (STH) energy conversion.

Cyanobacteria and green algae exhibit biological features that make them highly suitable for the engineering of hydrogenase based STH systems. They feature a highly specialized light harvesting machinery coupled to a complex photosynthetic electron transport chain (PETC). Briefly, protons (H^+^) and electrons are extracted from the water splitting reaction at photosystem II (PSII). Subsequently, H^+^ reduction to hydrogen can be performed by redirecting the high potential electrons via the PETC towards photosystem I (PSI) and then transferring them to a hydrogenase. Indeed, most cyanobacteria and green algae feature at least one native hydrogenase and certain strains are capable of native hydrogen production under specific conditions. Commonly, hydrogenases in cyanobacteria are used as a mechanism to recover energy from the H_2_ produced as a byproduct of nitrogenase in nitrogen fixing strains (uptake hydrogenases). Additionally, hydrogenases also act as photoprotective electron valves during periods when metabolic electron sinks are inactive or cannot cope with a sudden increase in the reducing power supplied from photosynthetic apparatus (bidirectional hydrogenases). Important differences between both organisms are found in the light harvesting systems and internal cellular organization. While eukaryotic green algae harbor light harvesting complexes similar to plants, cyanobacteria are prokaryotes and feature phycobilisomes containing specific light harvesting phycobiliproteins not present in green algae. Still, both exhibit photosynthetic efficiencies and biomass production rates significantly higher than any plants. Previous reviews (Khanna and Lindblad, 2015; Kosourov et al., 2021; Touloupakis et al., 2021; Li et al., 2022; Redding et al., 2022) provide a broad picture of the STH paradigm, reviewing the most relevant photobiological H_2_ production strategies reported to date.

Photobiological H_2_ production can occur via two main mechanisms, employing either nitrogenases or hydrogenases. The former relies in co-occurring H_2_ production during N_2_-fixation. Moreover, nitrogenase H_2_ production requires both ATP and reducing equivalents. Therefore, the maximum efficiency of nitrogenases is significantly lower than hydrogenase-based systems, which only require reducing equivalents from a compatible electron donor. Additionally, some hydrogenases have turnover numbers up to 1,000-fold greater than those of nitrogenases (Hallenbeck and Benemann, 2002). Thus, hydrogenases are undoubtedly better catalysts for the purpose of biotechnological STH conversion owing to its higher catalytic rates and more efficient energy utilization. Historically, hydrogenase derived H_2_ production has attracted less attention due to a range of limitations, including the bidirectional nature and catalytic bias of the enzymes, and an apparent trade-off between turnover rates and O_2_ tolerance. These perceived issues, in combination with an earlier lack of tools for extensive engineering of photosynthetic organisms and limited information about hydrogenase structure(s) and mechanism(s) has hindered the development of hydrogenase-driven STH production. However, during the last years, a number of multiple factors has unlocked the possibility of further development of hydrogenase based photobiological H_2_ production.

Firstly, a deeper understanding of structural and functional principles of hydrogenases has been obtained. In addition, several novel hydrogenases have been discovered or engineered, exhibiting highly interesting features for biotechnological utilization. Secondly, a wider understanding of photosynthetic metabolism, cell physiology and regulation has been acquired during the last decades, enabling the development of more sophisticated genetic engineering and synthetic biology tools. In combination with a growing interest in photosynthetic microorganisms within the scientific community and industry, this has unlocked the possibility to further engineer photosynthetic organisms for photobiological hydrogenase-driven H_2_ production. Current electrochemical STH technologies, feature energy conversion efficiencies ranging between 10 and 20% and production costs about 6 to 8 € · kg H_2_^−1^ (Grimm et al., 2020). Maximum theoretical estimations for non-engineered photobiological hydrogen production indicate that efficiencies close to 10% could be achieved, being on-par with current electrochemical technologies (Kruse et al., 2005). We expect that further developments combining the different strategies summarized here will allow a dramatic increase of efficiency relative to current approaches. Depending on the obtainable STH conversion efficiencies and further bioprocess design, our production costs estimations may be as low as 5 € · kg H_2_^−1^, making photobiological H_2_ production potentially cheaper than current STH electrochemical methods.

In the present review we provide a summary of the, in our opinion, most relevant achievements in the context of hydrogenase-based photobiological H_2_ production systems, highlighting how these efforts are bringing us closer towards the development of cost-effective photobiological hydrogen production technologies.

## 2. Hydrogenases overview

### 2.1. Hydrogenases for photosynthesis-coupled hydrogen production

Hydrogenases are an evolutionary convergent enzyme family of oxidoreductases that catalyze the reduction of protons into dihydrogen as well as the reverse oxidation of dihydrogen into two protons and two electrons. They are found in all domains of life, predominately present in Archaea and Bacteria and rarely in Eukarya (Vignais and Billoud, 2007; Greening et al., 2016). Hydrogenases are classified into [NiFe], [FeFe], and [Fe] hydrogenases based on their unique organometallic cofactors that facilitate reversible redox reactions (Lubitz et al., 2014; Berggren et al., 2022). [Fe] hydrogenases show comparably low catalytic rates and differ substantially from [NiFe] and [FeFe] hydrogenases in their reaction mechanism and substrates (Vogt et al., 2008). Therefore, they are not considered suitable for a photosynthesis-coupled hydrogen production and will not be discussed further. [NiFe] and [FeFe] hydrogenases on the other hand can achieve hydrogen turnover *in vivo* by using ferredoxin and NAD(P)H/NAD(P)^+^ (among others) as redox partner which are closely linked to PSI and the photosynthetic machinery (Appel et al., 2020; Lupacchini et al., 2021) However, we note that the they are extremely diverse enzyme families, reflecting large variations in activity between different hydrogenases (Land et al., 2020; Berggren et al., 2022).

Efficient (photo-)biological hydrogen production is dependent on successful expression of active and robust hydrogenases. The oxygen (O_2_) sensitivity of most [NiFe] and [FeFe] hydrogenases represent a particular challenge for coupling *in vivo* hydrogen production to the photosynthetic electron transport, as PS II produces molecular oxygen during the water oxidation. There are critical differences between hydrogenases in their reactivity towards O_2_, but the terminology of oxygen tolerance and oxygen sensitivity can lead to misconceptions.

In the following, we will distinguish between oxygen-sensitive and oxygen-insensitive hydrogenases. Oxygen-insensitive enzymes are defined as functional under atmospheric oxygen concentration. Conversely, oxygen-sensitive hydrogenases are inhibited by oxygen, but can be further subdivided into oxygen-sensitive tolerant and oxygen-sensitive intolerant hydrogenases based on the reversibility of their oxygen-induced inhibition. It should be noted that these definitions are different as compared to the general hydrogenase literature, where the term O_2_ tolerant is commonly used to describe what we would refer to as O_2_ insensitive hydrogenases (see, e.g., Lubitz et al., 2014). Most [NiFe] hydrogenases reported to-date are oxygen-sensitive tolerant enzymes, i.e., they display reversible oxygen-induced inhibition. A few [NiFe] hydrogenases even stand out as oxygen-insensitive as their function under atmospheric oxygen concentrations is not impaired (Buhrke et al., 2005; Leroux et al., 2008; Liebgott et al., 2010; Schäfer et al., 2013; Grinter et al., 2023). In contrast, the majority of [FeFe] hydrogenases are oxygen-sensitive intolerant enzymes as the active site is irreversibly damaged when exposed to trace amounts of oxygen. Still, there are also reports of oxygen-sensitive tolerant [FeFe] hydrogenases (Morra et al., 2016; Corrigan et al., 2020; Winkler et al., 2021). Within these broad classifications, the kinetics of the inactivation and reactivation vary between enzymes (see for example Cracknell et al., 2008; Goldet et al., 2009; Lukey et al., 2011; Kubas et al., 2017; Koo and Swartz, 2018).

Most [NiFe] hydrogenases are dimeric enzymes, consisting of a “small” and a “large” subunit of which the latter one contains the active site with the catalytic [NiFe] cofactor. The small subunit harbors usually a chain of three FeS-clusters, denoted as proximal, medial and distal depending on their relative position to the [NiFe] active site, which ensures electron transport between protein surface and NiFe center. The medial cluster is often [3Fe-4S] while the proximal and distal clusters are [4Fe-4S] (Volbeda et al., 1995, 1996). Exceptions to this FeS-cluster arrangement have been observed, and include the soluble bidirectional cytoplasmatic [NiFe] hydrogenases found in different aerobic microorganisms such as the HoxFUYH complex from *Synechocystis* PCC 6803. The NAD(P)^+^-reducing [NiFe] hydrogenase HoxFUYH is a multi-subunit complex with HoxH as catalytic (or “large”) subunit. The accessory subunits HoxFUY build a complex electron relay where HoxY serves as the immediate electron transfer partner of HoxH, and can be considered equivalent to the small subunit, but features only a single [4Fe-4S] cluster (Carrieri et al., 2011). Other reported exceptions with changes to the electron relay clusters have been shown to influence oxygen tolerance and catalytic bias as further outlined below. [FeFe] hydrogenases often show substantially higher turnover rates than [NiFe] hydrogenases at the expense of being irreversibly inactivated by trace amounts of molecular oxygen (Goldet et al., 2009). [FeFe] hydrogenases are generally functional as monomers but can consist of several domains (Calusinska et al., 2010; Land et al., 2020). The H-domain is essential for the function as it contains the active site (H-cluster) which comprises a canonical [4Fe-4S] cluster ([4Fe-4S]_H_) that is linked to the di-iron metal center via a bridging cysteine. [FeFe] hydrogenases commonly carry additional domains with FeS-clusters in the N- or C-terminus of the H-domain (Meyer, 2007). The modularity of these so-called F-domains ensures that they can provide the enzyme with different functions, e.g., ensuring efficient intra- or intermolecular electron transport between the highly conserved active site and redox partners (Gauquelin et al., 2018).

Even though [NiFe] and [FeFe] hydrogenases are not evolutionary related, they share certain key characteristics among their active sites. Both catalytic cofactors are dinuclear, and the metal ions bridged by thiol ligands. In the case of [NiFe] the thiols are derived from cysteine, while [FeFe] hydrogenases are dependent on a 2-aza-propane-dithiolate (adt) ligand that bridges the di-iron metal center. Furthermore, both feature Fe ions ligated by carbon monoxide and cyanide. These strong field ligands keep the Fe ions in a low-spin and low valent state, which enables the heterolytic splitting of H_2_ or the dihydrogen formation. Considering their unique structures, the assembly and introduction of these organometallic active site cofactors unsurprisingly depend on hydrogenase-type specific maturation enzymes (Lubitz et al., 2014). Cyanobacteria express natively one or two [NiFe] hydrogenases, which either serve as sink for excess electrons from photosynthesis or are closely linked to the hydrogen uptake for nitrogenase activity. They rely on the expression of at least six [NiFe] cofactor maturation enzymes (HypABCDEF) which have been identified in *E. coli* but are known to be existent in all organisms that express [NiFe] hydrogenases. [FeFe] hydrogenases on the other side are not present in any known cyanobacterial organism. So far [FeFe] hydrogenases and their specific cofactor maturation enzymes (HydEFG) have been exclusively found in algal organisms and non-cyanobacterial prokaryotes (Böck et al., 2006; Khanna and Lindblad, 2015). Consequently, heterologously expressed hydrogenases are only functional if either the expression host expresses natively the specific maturation enzymes or the maturation machinery is co-expressed (King et al., 2006; Wegelius et al., 2021). It was recently shown that maturation enzymes of [FeFe] hydrogenases require an additional auxiliary pathway providing a co-substrate, a glycine cleavage system generating methylated lipoyl-H-protein (Pagnier et al., 2022). However, this co-substrate may usually be regenerated by the native host metabolism.

### 2.2. Engineering of [Fe-Fe] hydrogenases

Several different approaches have been explored to achieve a photosynthesis-coupled hydrogen production. In the most cases, the work focused on coupling hydrogenases to electron carriers or PSI itself. Even though the high catalytic rates of [FeFe] hydrogenases exhibit a considerable potential for such applications the oxygen sensitivity and the irreversible inactivation are major obstacles as mentioned above. Therefore, it is necessary to explore possible ways to create oxygen-insensitive [FeFe] hydrogenases.

In the case of [NiFe] hydrogenases evolution has provided a number of different features identified to provide oxygen tolerance. Primarily these protection strategies appear to focus on the controlled reduction of O_2_ to avoid reactive oxygen species (ROS) and/or limiting gas access to the O_2_-sensitive active-site. These features could be considered for the generation of oxygen-insensitive [FeFe] hydrogenase. For several membrane bound oxygen-insensitive (originally referred as oxygen tolerant) [NiFe] hydrogenases an altered FeS cluster has been reported proximal to the di-nuclear metal center. This proximal [4Fe-3S]-6Cys cluster enables the oxygen-insensitive [NiFe] hydrogenases to rapidly transfer two electrons to the active site, ensuring detoxification of O_2_ through reduction to water (Ogata et al., 2016). However, such an alteration of the [FeFe] active site is likely challenging as the proximal [4Fe-4S]_H_-cluster is directly involved in the catalytic cycle. However, there are reports of F-clusters improving O_2_ tolerance (Kubas et al., 2017). Still, we note that any protection mechanism depending on reduction of O_2_ will result in loss of electrons towards unproductive process. Several studies also explored oxygen tolerance achieved by limiting mass transfer through the putative gas channels (Buhrke et al., 2005; Leroux et al., 2008; Liebgott et al., 2010). Buhrke et al. reported that in the H_2_-sensing [NiFe] hydrogenase RH from *Ralstonia eutropha* bulky hydrophobic residues restrict the diffusion of molecular oxygen to the active site. A recent striking example of this mechanism at play is provided by the Huc hydrogenase from *Mycobacterium smegmatis*, which is capable of performing H_2_ oxidation under fully aerobic conditions (Grinter et al., 2023). Molecular dynamics experiments of [FeFe] hydrogenases suggested that O_2_ can only diffuse through hydrophobic gas channels whereas H_2_ can also diffuse freely through temporary cavities in the flexible protein structure (Cohen et al., 2005). However, engineering approaches which aimed at modulating the gas transfer through the assumed main gas channel have not been successful so far (Lautier et al., 2011). Furthermore, a complete obstruction of the gas channels in [FeFe] hydrogenases is also not assumed beneficial, as the build-up of H_2_ accumulation in the active site can inhibit the H_2_ production (Fourmond et al., 2013).

The main protection mechanism against O_2_-induced degradation of the [FeFe] hydrogenase cofactor identified to-date involves binding of thiol ligands at the open Fe-coordination site involved in catalysis. For sulphate-reducing bacteria *Desulfovibrio desulfuricans* and *Desulfovibrio vulgaris* Hildenborough, and their respective [FeFe] hydrogenases *Dd*HydAB and *Dv*HydAB, it has been shown that an extrinsic sulfide group can coordinate the substrate binding site. This protected state of the active site causes reversible inactivation but also oxygen-sensitve tolerant characteristics (Roseboom et al., 2006). Chemical treatments of purified [FeFe] hydrogenases with NaS_2_ can induce this state *in vitro* under oxidizing conditions (Rodríguez-Maciá et al., 2018). More recently, a similar effect was reported through the binding of exogenous cyanide (Martini et al., 2023). However, in contrast to thiols, cyanide binding appears irreversible thus also causing inactivation of the enzyme. Up to now only one intrinsically oxygen-sensitive tolerant [FeFe] hydrogenase from *Clostridium beijerinckii* (*Cb*A5H/*Cb*HydA1) has been identified and characterized by Morra et al. (2016). An active site neighboring cysteine enables a reversible inactivation of the [FeFe] hydrogenases by coordinating the substrate binding site of the H-cluster with its thiol sidechain (Corrigan et al., 2020; Winkler et al., 2021). This coordination prevents the reduction of O_2_ at the H-cluster and therefore also the formation of reactive oxygen species that could irreversibly damage the active site cofactor. Winkler et al. also reported that the interaction of individual distant residues around the loop region of the protective cysteine are crucial for an oxygen-protective conformation. In another recent study it was shown that one amino acid exchange on the surface of *Cb*HydA1 can modulate the transition rates between the inactivated and oxygen-sensitive state (Rutz et al., 2023). With the above-mentioned findings it stands out that protein engineering of characterized [FeFe] hydrogenases is of significant importance to develop more robust hydrogen-producing biocatalysts. In parallel, the identification and characterization of new [FeFe] hydrogenases are needed to explore more beneficial properties such as oxygen sensitivity (Morra, 2022).

Another aspect to consider when using hydrogenases is that they are bidirectional catalysts. However, the activity can be influenced by many parameters, and often the enzymes show a preference for either hydrogen production or oxidation. This kinetic discrepancy between the forward and reverse reaction is referred to as “catalytic bias.” Amino acid side chains in the first coordination sphere of the organometallic cofactor can influence the catalytic bias (Land et al., 2020). Additionally, when present, the FeS clusters in the F-domains of [FeFe] hydrogenase have been shown to introduce a strong catalytic bias (Pandey et al., 2017; Birrell et al., 2021).

## 3. Improving photobiological H_2_ production. Non-native hydrogenases and photosynthesis re-engineering in oxygenic photosynthetic microorganisms

Engineering efforts towards efficient photobiological H_2_ production has focused into two main complementary paths. First, removing native hydrogenases capable of H_2_ uptake and optimizing the hydrogenase as the key component of the H_2_ production system. In this context, heterologous, chimeric, or fully synthetic hydrogenases with improved features are over-expressed as a way to enhance hydrogen production. Importantly, enzyme expression also needs to be at high levels, as it has been documented that hydrogen production correlates with the abundance of active hydrogenase enzyme *in vivo* (Weiner et al., 2018). Secondly, significant efforts have been devoted at re-engineering different sub-systems within the complex bioenergetic machinery of PETC and/or light harvesting systems, aiming to enhance the overall electron supply and efficiently channel it towards the expressed hydrogenase.

### 3.1. Expression of non-native hydrogenases in cyanobacteria

During the last decades a deep understanding of cyanobacterial physiology has been acquired, giving rise to the development of an expanding synthetic biology toolbox for sophisticated strain engineering. These factors in combination with its generally faster growth rates, more flexible light harvesting machinery and engineering simplicity when compared to green algae makes them a very promising platform for developing highly efficient green cell H_2_ producing factories. Table 1 provides a summary of achievements on H_2_ production when expressing non-native hydrogenases in oxygenic photosynthetic microorganisms.

**Table 1:** Heterologous expression of hydrogenases in cyanobacteria and green algae.

Host	Implemented Strategy	Outcome and conclusion/Key findings	Reference
Cyanobacteria			
*Synechococcus elongatus* PCC 7942	IPTG- inducible heterologous expression of [FeFe] hydrogenases (HydA) and psba1 controlled expression of [FeFe] maturation machinery, *in vivo*	2.8 μmol H_2_ · h^−1^· mg Chl*a*^−1^, ferredoxin-hydrogenases compatibility is crucial for efficient coupling to native redox metabolism	Ducat et al. (2011)
*Anabaena/Nostoc* PCC 7120	Heterologous expression of [FeFe] hydrogenase operon from *Shewanella oneidensis* MR-1 controlled by heterocyst-specific promoter *phetN*, deletion of native NiFe hydrogenases, *in situ*	1 μmol H_2_ · h^−1^· mg Chl*a*^−1^in *in situ* experiments (supplemented with dithionite and MV)	Gärtner et al. (2012)
*Anabaena/Nostoc* PCC 7120	Heterologous expression of [FeFe] hydrogenases (HydA) and maturation machinery in heterocyst controlled by late-phase promoter, cyanoglobin expression under control of *patB* promotor *in vivo*	400 μmol H_2_ · mg Chl*a*^−1^ under argon atmosphere 13 μmol H_2_ · mg Chl*a*^−1^ under aerobic conditions O_2_ level actively lowered by cyanoglobin GlbN in heterocyst, competition of nitrogenase and hydrogenase for reduced ferredoxin	Avilan et al. (2018)
*Synechocstis* PCC 6803	Expression of fusion protein PsaD-HoxYH (native NiFe hydrogenases and PSI subunit), deletion of *hoxEFUYH* and *psaD*	17-fold increase in H_2_ concentration (546 μM) compared to WT. Higher temporary H_2_ production rates than WT. H_2_-uptake circumvented by avoiding backwards electron transfer to PSI. Limited rate of H_2_ produced from oxygenic photosynthesis	Appel et al. (2020)
Green algae			
*Chlorella* sp. DT	Heterologous overexpression of clostridial [FeFe] hydrogenase (HydA) under control of two different promoters in aerobic and sulfur-supplied conditions	Native maturation of heterologous [FeFe] hydrogenase, H_2_ production 10-fold higher than in WT strain, not proportional increase with protein expression level	Chien et al. (2012)
*Chlamydomonas reinhardtii*	Expression of a Fd-HydA1 fusion protein under control of the *psaD* promoter	Enhanced electron flow from PSI, leading to 4.5-fold higher hydrogen production than WT at low protein expression levels. Increased oxygen tolerance of the [FeFe] hydrogenase, probably due to Fd protection	Eilenberg et al. (2016)
*Chlamydomonas reinhardtii*	Screening for *Chlamydomonas reinhardtii* mutants overexpressing a Fd-HydA1 fusion integrated either in nuclear genome (*psaD* promoter) or chloroplasts chromosome (*psaA* promoter)	Sustained H_2_ production that correlates linearly with active enzyme abundance. Enzyme showed 4.5-fold increase in hydrogenase activity compared to WT, even at high expression levels. Fd fusion limited hydrogenase activation, where up to 85% of the overexpressed hydrogenase chimera remained in the apo-form	Weiner et al. (2018)
*Chlamydomonas reinhardtii*	Expression of two different Fd-HydA1 chimeric proteins harboring a mutant Fd (D19A, D58A) with limited affinity towards FNR under a sulfur-limitation induced promoter	Chimeric protein expressed at comparable levels of HydA1 in WT. The Fd-HydA1 harboring a 25 aa linker exhibited better performance. Average production of 111.7 ± 3.4 ml · H_2_ L^−1^, a 4.6-fold increase in H_2_ production compared to WT	Xiong et al. (2021)
*Chlamydomonas reinhardtii*	Design of a PSI-hydrogenase chimera by. Fusing the stromal PsaC subunit to either HydA1/HydA2 hydrogenases and *in vivo* expression in a Δ*psaC Chlamydomonas reinhardtii* mutant strain	Fusion chimeras were successfully activated by HydEFG maturases (>90% active protein), additionally, up to 60% of O_2_-inactivated PSI-HydA chimeras could be reactivated after reducing O_2_ concentration. Enhanced H_2_ production was observed, mainly derived from indirect PETC regulation, lowered LET and O_2_ production Average production of 14.0 ± 1.7 μmol H_2_ · h^−1^· mg Chl*a*^−1^ (for PSI-HydA2 chimera, expressed at 7 times lower levels than WT PSI, leading to reduced PSII activity and enabling sustaining H_2_ production due to limited O_2_ buildup). PSI-HydA1 chimera exhibited only 50% of PSI-HydA2 turnover rate. Its expression were 5 times higher than PSI-HydA2, restoring close-to-WT levels of PSII activity an increased O_2_ production, leading to a 10-fold overall H_2_ production compared to PSI-HydA2 chimera	Kanygin et al. (2020, 2022)

#### 3.1.1. Expression and activation of [FeFe] hydrogenases in cyanobacteria

[FeFe] hydrogenases have been widely considered the most active and promising group for hydrogenase-catalyzed H_2_ production. Unfortunately, cyanobacteria do not feature native [FeFe] hydrogenases, neither the maturation machinery required for its activation. This limitation has been overcome by the co-expression of an [FeFe] hydrogenase together with its maturation machinery (HydEFG). An [FeFe] hydrogenase from *Clostridium acetobutylicum* (HydA1) together with the HydEF and HydG maturation machinery from *Chlamydomonas reinhardtii* were introduced and expressed in the non-N_2_-fixing unicellular cyanobacterium *Synechococcus elongatus* PCC 7942 (Ducat et al., 2011). The introduced enzyme showed an *in vivo* hydrogenase activity which connected to the light-dependent reactions of PETC under anoxic conditions. The heterologous hydrogenase supported limited growth in light using H_2_ as the sole source of reducing equivalents. Moreover, the importance of adequate coupling between the non-native hydrogenases and the native electron carriers was noted, where an expression of a non-native ferredoxin improved the H_2_ production. Comparably, the native [FeFe] hydrogenase operon from the bacterium *Shewanella oneidensis* MR-1 was expressed in heterocysts of the filamentous cyanobacterium *Nostoc* PCC 7120, leading to the assembly of an active enzyme (Gärtner et al., 2012). In a similar fashion, the native maturation machinery and of hydrogenase HydA of *Clostridium acetobutylicum* has been reported to yield an active [FeFe] hydrogenase in *Nostoc* PCC 7120 using the heterocyst specific *nifH* promoter. Furthermore, by co-expressing a GlbN cyanoglobin from *Nostoc commune* to decrease the oxygen levels, *in vivo* H_2_ production was observed concomitantly with oxygenic photosynthesis in the vegetative cells of the filaments (Avilan et al., 2018). Earlier reports of active [FeFe] hydrogenases in cyanobacteria without the introduction of the specific [FeFe] hydrogenase maturation machinery (Asada et al., 2000; Berto et al., 2011) requires further confirmations.

However, it is possible to circumvent the biological maturation of [FeFe] hydrogenase by using a semi-synthetic activation approach. This procedure consists on the heterologous expression of the inactive [FeFe] apo-hydrogenase in living cells, followed by subsequent incubation with a synthetic [2Fe]_H_ subcluster mimics, leading to the formation of an active H-cluster and rendering a mature holo-[FeFe] hydrogenase. Semi-synthetic [FeFe] hydrogenase maturation was first demonstrated in *E. coli* (Khanna et al., 2017). The mechanism by which the synthetic cofactor enters the cell and activates the enzyme remains to be elucidated, but it has been found to occur on a minute-time scale (Mészáros et al., 2018). Later work expanded its utilization to cyanobacteria (Wegelius et al., 2018, 2021). The resulting non-native, semisynthetic enzyme unmistakably links to the native metabolism of the photosynthetic cell and retain its H_2_ production capacity for several days with an activity based on availability of electrons.

To date studies on [FeFe] hydrogenase expression in cyanobacteria has focused almost exclusively on representative examples of “prototypical” Group A [FeFe] hydrogenases. However, Wegelius et al. also reported on the variance in H_2_ production arising from employing [FeFe] hydrogenases from different phylogenetic groups (Wegelius et al., 2021), and showed that the Group D enzyme *Thermoanaerobacter mathranii* HydS displayed distinct differences in its hydrogen gas production profile relative to the Group A enzymes from *C. reinhardtii* and *Solobacterium moorei*.

#### 3.1.2. Expression of [NiFe] hydrogenases and hydrogenase fusion constructs in cyanobacteria

Heterologous expression of [NiFe] hydrogenases provides the possibility to take advantage of the native cyanobacterial [NiFe] hydrogenase maturation machinery. Thus, it circumvents the challenge of efficiently co-expressing the hydrogenase, together with its maturation machinery (HydEFG) required to obtain active [FeFe] hydrogenases. As such, it provides a potentially more straightforward way to study the effect of introducing enzymes with improved properties or to engineer fusion proteins between the native cyanobacterial [NiFe] hydrogenase and different components of the PETC, which in turn can guide efforts related to [FeFe] hydrogenase.

Early research reported the expression of heterologous [NiFe] hydrogenases from *Alteromonas macleodii* and the bidirectional [NiFe] hydrogenase from *Thiocapsa roseopersicina* in a mutant *Synechococcus elongatus* PCC 7942 lacking the native bidirectional hydrogenase structural genes (ΔHoxYH). Although the proteins did not show any activity *in vivo*, *in vitro* H_2_ production confirmed the successful activation of the two hydrogenases, and the coupling of the catalytic subunits (HoxYH) from *T. roseopersicina* to the native diaphorase HoxEFU subunits. However, cloning of multiple hydrogenase accessory genes from native organisms was still required to achieve the assembly and activation of functional [NiFe] hydrogenases, highlighting the diversity of hydrogenase maturation mechanisms even within the realm of [NiFe] hydrogenases (Weyman et al., 2011).

In similar endeavors, O_2_-tolerant, and NAD(H)-dependent hydrogenase from *Ralstonia eutropha* (*Re*SH) was expressed in *Synechocystis*PCC 6803 featuring a knockout of the native hydrogenase (Lupacchini et al., 2021). In this study, the engineered strain was able to continuously produce H_2_ under illumination at a higher rate than the wild-type (WT) and for up to 20 h under illumination. This result indicates that the native maturation machinery was sufficient to mature the heterologously expressed [NiFe] hydrogenase without the requirement of its accessory *hyp*-genes. The sustained H_2_ production was attributed with the higher O_2_ tolerance of the *Re*SH. In addition, the *Re*SH expressing strain was able to use H_2_ as sole electron source, enabling autotrophic growth, even when water oxidation at PSII was blocked by DCMU (3-(3,4-dichlorophenyl)-1,1-dimethylurea) addition. This indicates clear coupling between the introduced hydrogenase and the native cyanobacterial redox metabolism. However, due to the NAD(H) dependent nature of *Re*SH, a high intracellular NADH/NAD^+^ ratio and efficient NADH supply are required for H_2_ production. This was achieved by glucose supplementation and blocking of any other metabolic electron sinks such as nitrate or carbonate/CO_2_ assimilation, where the cells were unable to produce H_2_ in the absence of both conditions. In the absence of glucose supplementation and presence of the native electron sinks for assimilation pathways no H_2_ production was observed. Overall, these results demonstrate the possibility of expressing heterologous O_2_-tolerant hydrogenases in cyanobacteria, and how the native cyanobacterial maturation machinery can activate heterologous [NiFe] hydrogenases. Nonetheless, it is also clear that further engineering of the electron channeling towards the hydrogenase is required for the development of an efficient H_2_ production system.

A complementary strategy is focused on enhancing the electron supply to the hydrogenase. PSI-hydrogenase fusion constructs have been considered an appealing solution. Critically this design could allow to intercept electrons from the PETC before they reach the Fd pool, thus avoiding the need for extensive engineering of hydrogenase coupling with the redox metabolism and competition with other native electron sinks. Following this principle, light-driven H_2_ production from the *Desulfovibrio vulgaris* [NiFe] hydrogenases fused to cyt-c3 and PsaE subunit of PSI was reported *in vitro* (Ihara et al., 2006). This study highlighted the suitability of stromal PSI subunits as targets for PSI-Hydrogenase fusion proteins, as well as the importance of considering electron transport pathways from PSI to the fused hydrogenase moiety. More interestingly, the native [NiFe] hydrogenase of *Synechocystis* PCC 6803 (SynH) was fused to PSI PsaD subunit and overexpressed in a *Synechocystis* PCC 6803 mutant lacking the native hydrogenase (Appel et al., 2020). To date, this study reported the highest headspace H_2_ concentration achieved using *Synechocystis* PCC 6803 (> 500 μM) and the only report of H_2_ production *in vivo* using a PSI-hydrogenase fusion construct. The study shows how in spite of the reversible nature of SynH, H_2_ uptake is virtually avoided in the engineered PSI-SynH system. This indicate that on the contrary to previous hydrogenase expression strategies, PSI-Hydrogenase fusion proteins can alter the enzyme’s native bias by impeding reversed electron flow from the hydrogenase moiety back to PSI. Additionally, the study addressed the relevance of the PsaD-SynH fusion protein design, aiming to place the exposed external [4Fe4S] cluster of *Synechocystis* PCC 6803 native hydrogenase closer to the F_B_ and F_A_ [4Fe4S] clusters of the PSI PsaC subunit. The expressed SynYH hydrogenase corresponds only to the catalytic subunits of the native [NiFe] hydrogenase, reducing its ability to interact with native electron donors such as ferredoxin, thus reducing the possibility of hydrogen oxidation. The observed results indicate that a direct electron transfer from the [4Fe4S] clusters of the PSI PsaC subunit towards the [4Fe4S] cluster of the hydrogenase is the main, if not the only, electron source for the catalytic activity. These findings open the possibility of further engineering PSI-Hydrogenase systems where hydrogenases with desirable features are fused to PSI, as well as developing new fusion constructs with improved electron transfer properties. The latter could be achieved by reducing the distance between the PsaC [4Fe4S] donor clusters and the acceptor [4Fe4S] cluster of the hydrogenase (Appel et al., 2020) as well as via the introduction of electron carriers within the fusion construct’s structure (Walters and Golbeck, 2020; Günzel et al., 2022). Similarly, coupling of the PSI PsaC subunit’s F_B_ [4Fe4S] cluster to a *Clostridium acetobutylicum* HydA using a dithiol-based molecular wire has been reported (Lubner et al., 2011). The resulting biological/organic nanoconstruct exhibited a 2-fold increase in overall photosynthetic electron turnover in PSI and high light-driven H_2_ production using an *in vitro* assay. These results demonstrate the relevance of PSI-Hydrogenase fusions as a promising way to overcome diffusional limitations of soluble redox carriers as well as limiting competition with other competing pathways. Undoubtedly, recent discoveries about hydrogenases’ interactions with native electron carriers will help guiding more rational engineering designs (Rumpel et al., 2015).

It worth noting that these studies highlighted that their reported photobiological H_2_ production titers where achieved in controlled experimental conditions avoiding O_2_ inactivation. In this context, electrons are normally obtained from other sources of reducing equivalents (mainly glucose) and transferred to the PETC via fermentative pathways (Appel et al., 2020; Lupacchini et al., 2021). These findings accentuate the relevance of combining the described engineering strategies with further engineering in order to remove the limitations imposed by O_2_ production in PSII.

### 3.2. Expression of hydrogenases and hydrogenase fusion constructs in green algae

Green algae harbor natively highly active [FeFe] hydrogenases and the associated HydEFG maturation machinery. Early studies of hydrogenase-driven H_2_ production in green algae traditionally focused on developing cultivation protocols that shifts cell metabolism towards anoxygenic conditions thereby inducing H_2_ production using nutrient-depleted media, as well as controlled pulse-illumination procedures (Melis et al., 2000; Kosourov et al., 2018). However, the growing availability of engineering tools developed during the last decade has led to several non-native hydrogenase expressions and other hydrogenase engineering strategies reported in green algae with promising results. Table 1 provides a summary of achievements on H_2_ production when expressing non-native hydrogenases in oxygenic photosynthetic microorganisms.

Chien et al. reported an up-to 10-fold increase in H_2_ production in engineered *Chlorella* DT strains. This was achieved by cloning and overexpression its principal native [FeFe] hydrogenase (Chien et al., 2012). Other engineering efforts focused on enhancing the redox coupling of the hydrogenase to PSI, aiming to reduce competition for PSI with Ferredoxin-NADP^+^ reductase (FNR). A synthetic ferredoxin (Fd) - [FeFe] hydrogenase protein was designed by fusing native Fdx1 and HydA from *C. reinhardtii*. Strains expressing the fusion constructs showed 4.5-fold increase in specific H_2_ production and surprisingly an enhanced O_2_ tolerance (Eilenberg et al., 2016). A later report studied in deeper detail the *in vivo* performance of the Fdx1-HydA1 synthetic protein and aimed to enhance H_2_ production by screening mutants overexpressing the fusion construct. Developed strains exhibited higher protein expression levels and longer-lasting sustained H_2_ production. However, this study also reported that up to 85% of the synthetic Fdx1-HydA1 fusion remained in the inactive apo-protein form, indicating inefficient *in vivo* hydrogenase maturation (Weiner et al., 2018). These findings highlight the importance of further synthetic protein engineering to optimize not only protein expression but also allow efficient hydrogenase maturation. More recently, a mutant ferredoxin with reduced electron transfer to FNR (Fdx1 D19A D58A) was fused to HydA1 using two different, 15 or 25 amino acids (aa), linkers. The obtained strains overexpressing the fusion constructs showed up to a 4.6-fold increase in H_2_ production when compared with a strain expressing only HydA1 at comparable levels (Rumpel et al., 2014).

Similarly, to studies in cyanobacteria, PSI-hydrogenase fusion constructs have been expressed in green algae, leading to enhanced *in vivo* H_2_ evolution. These efforts have primarily focused on *C. reinhardtii* which contains two native [FeFe] hydrogenases commonly denoted as HydA1 and HydA2. *In vitro* studies have suggested that HydA1 is primarily involved in H_2_ production, while HydA2 appears more biased towards H_2_ oxidation (Hambourger et al., 2008). In these studies, the stromal PsaC subunit of PSI was fused to *C. reinhardtii* HydA2. The engineered strain showed fermentative H_2_ production, reaching approximately 60% of WT activity in anoxic dark conditions, indicating that the HydA2 moiety was still able to accept electrons from Fd. Furthermore, the strain showed a 7-fold lower abundance of the PSI-HydA2 construct compared to PSI in wild-type cells. The reduced PSI pool lead to a downregulation of native PSII activity which alters PETC regulation, mainly constraining the LET and consequently reducing the O_2_ evolution. This lowered O_2_ production may reduce the hydrogenase inactivation thereby increasing observed H_2_ production. Overall, the expression of PSI-HydA2 fusion allowed a sustained H_2_ production upon illumination at rates comparable to the transient H_2_ production of the WT, arguably mainly due to the changes in PETC regulation. More recently, the potentially more efficient HydA1 was selected and a PSI-HydA1 fusion protein was designed and expressed in *C. reinhardtii* with the aim to increase H_2_ production (Engelbrecht et al., 2021). The obtained strain accumulated 5-times more PSI-HydA1 protein than the previous PSI-HydA2 strain and exhibited a comparable interaction with native Fd in dark anoxic conditions. However, the turnover frequency of the PSI-HydA1 chimera turned out to be 50% of the previous PSI-HydA2 construct, and overall H_2_ production by the fusion protein was 10-times lower than the previous PSI-HydA2 strain. These activities appear to conflict with the reported intrinsic biases of HydA1 of HydA2 (Engelbrecht et al., 2021). However, the results can largely be rationalized to higher abundance of PSI in the PSI-HydA1 fusion protein strains, releasing the constrains on PSII-driven O_2_ evolution. Furthermore, the report shows how the native HydEFG machinery was able to activate the apo-PsaC-HydA1 chimera expressed before the induction of anoxic conditions, reaching turnover rates above 90% of the fully active form. Additionally, it was also shown that upon O_2_ inactivation PSI-HydA1, the active site can be reinserted into the same PSI-HydA1 chimera by the maturases, inducing up to 60% of original hydrogenase activity recovery after 4 h of anaerobic adaptation (Kanygin et al., 2020, 2022).

It should be noted that in contrast to the [NiFe] hydrogenase-PSI fusion expressed in cyanobacteria (Appel et al., 2020), which was unable to produce H_2_ under dark fermentative conditions, the fusion constructs expressed in green algae (Kanygin et al., 2020, 2022) exhibited H_2_ production in darkness. Thus, it is unclear which is the main electron source of the hydrogenase. The reported dark fermentative H_2_ production of the PSI-HydA1/2 engineered strains indicate that the ferredoxin docking site is probably still free in the fusion protein, allowing the hydrogenase to accept electrons from ferredoxin. Consequently, the enhanced H_2_ production could be caused from the localization of the hydrogenase moiety in the proximal environment of PSI, thereby improving its access to reduced ferredoxin. Alternatively, it is also possible that the hydrogenase accepts electrons directly from the FeS cluster F_B_ of the proximal PsaC subunit, or even a combination of both formerly described mechanisms. Overall, in the case of PSI-hydrogenase fusions, proximity to the F_B_ [4Fe4S] cluster of the PSI PsaC subunit has been shown to be a critical requirement. The closeness to PSI ensures higher interaction with the reduced FeS clusters of the PsaC subunit or Fd and limits FNR coupling to PSI, broadening electron supply from Fd towards the hydrogenase (Moal and Lagoutte, 2012; Xiong et al., 2021).

### 3.3. Photosynthesis re-engineering strategies for improved photobiological H_2_ production

**Figure 1:**
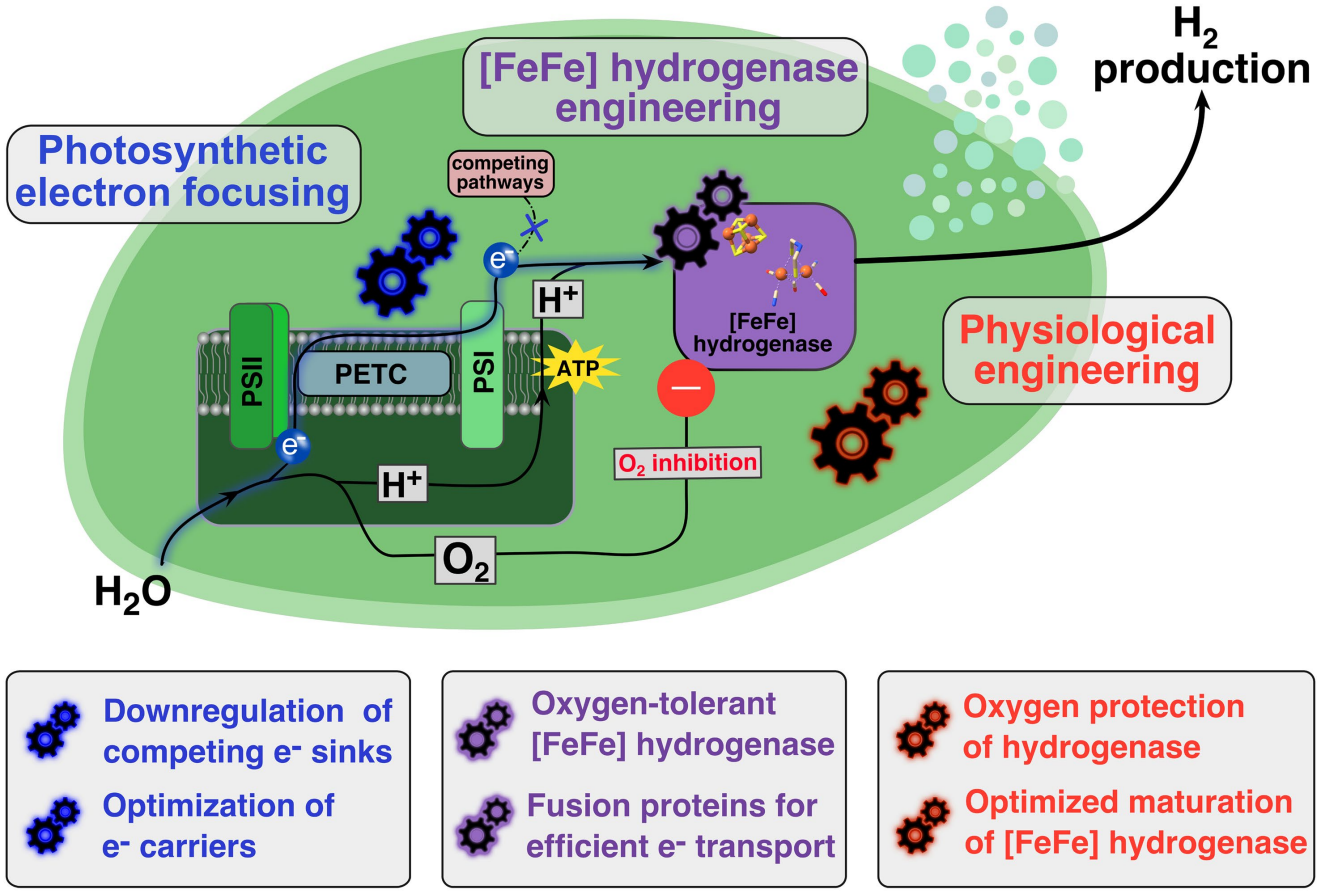
Schematic overview of engineering targets in photoautotrophic prokaryote for establishing efficient *in vivo* hydrogen production. Upon light-induced water splitting at photosystem II (PSII) oxygen is released and electrons are transferred *via* the photosynthetic electron transport chain (PETC) to photosystem I (PSI) where a cytoplasmatic electron carrier accepts these electrons. The photosynthetically-driven proton buildup in the thylakoids is enabling the ATP synthesis *via* ATP synthases at the thylakoid membrane. A heterologously expressed [FeFe] hydrogenase (purple box) uses the photosynthetically generated electrons and protons as substrates for a photobiological hydrogen production. To establish an efficient production system several features need to be targeted or introduced. Firstly, through photosynthetic electron focusing (blue gearwheels) the electrons transport from the photosynthetic machinery to the [FeFe] hydrogenase needs to be optimized. Secondly, the heterologously expressed [FeFe] hydrogenase has to be engineered (purple gearwheels) to work efficiently in the *in vivo* context. Especially, the oxygen-induced inhibition needs to be addressed by finding and introducing oxygen-insensitive or at least oxygen-sensitive tolerant enzyme variants into the *in vivo* system. Thirdly, physiological engineering efforts (red gearwheels) are necessary to assure that expressed [FeFe] hydrogenases can be activated and stay active for an extended period of time, as well as implementing further physiological modifications to protect the expressed hydrogenase from O_2_.

Herein we describe the most relevant strategies and breakthroughs that has been reported to increase photobiological H_2_ production beyond the expression of hydrogenases. In spite of the diversity of the different strategies, they can be grouped into three main focus areas: Light harvesting engineering, electron transport rewiring, and intracellular O_2_ concentration reduction. Figure 1 provides a summary of discussed strategies and breakthroughs to increase photobiological production beyond the expression of hydrogenases.

#### 3.3.1. Re-engineering light harvesting

As the ultimate driver of photobiological H_2_ production is light energy, enhancing the cells ability to harvest and utilize light more efficiently could enhance the electron availability for hydrogen production. The main strategies have focused on three directions: Broadening the light spectra utilization, reducing the light harvesting antennas for efficient light distribution and stronger illumination tolerance as well as optimizing the electron transport chain and reducing photoprotective energy dissipation.

##### 3.3.1.1. Introducing far-red chlorophylls

The far-red longest-wavelength absorbing chlorophyll known to date is chlorophyll F (Chl*f*). A Chl*f* synthase gene (*chlF*) of *Fischerella thermalis* PCC 7521 was heterologously expressed in *Synechoccocus elongatus* PCC 7002, leading to the incorporation of Chl*f* into the native PSI, functionally connected to the other pigments in the complex. Mutant strains lacking PSII and grown in far-red light achieved up to 50% of the Chl*f* content found in native far-red acclimated PSI. Although, the heterologous Chl*f* molecules were not as red-shifted as in their native environment, the assembly of a reduced amount of Chl*f* allowed to extend the photosynthetically active radiation up to 750 nm (Tros et al., 2020).

##### 3.3.1.2. Truncation of light harvesting complexes

Light-harvesting complexes have evolved to maximize solar energy capture. However, under strong illumination, the light-harvesting machinery absorb photons beyond the limits of photosystems capacity. Furthermore, large light-harvesting complexes also increase the shading effects in photobioreactors, causing to uneven light transfer.

It has been reported that truncation of light harvesting complexes in *Synechocystis* PCC 6803 leads to higher biomass accumulation and glycogen production while maintaining growth rates comparable to the WT. The effects were similar for deletion of phycocyanin (*Δcpc*) (Lea-Smith et al., 2014) or a phycobilisomes anchor protein (*ΔapcE*) (Joseph et al., 2014). Similar truncation strategies has also been reported to increase photosynthesis efficiency and biomass accumulation in green algae, extensively reviewed by Kumar et al. (2021). The potential influence of mutant cyanobacterial strains with unanchored phycobilisomes (*ΔapcE*), phycocyanin knockout (*Δcpc*), or whole phycobilisomes deletion (PAL mutant) have been studied. Overall antennae truncation lead to more resistant strains against light stress as well as reduced energy requirements for antenna synthesis and PSII repair. Additionally, mutants exhibited physiological adaptations upregulating linear electron transport (LET) and enhanced PSII expression and enhanced PSI turnover (Bernát et al., 2009). Nonetheless a higher number of PSII centers also leads to increased O_2_ evolution, an effect that should be considered when coupling LET to O_2_-sensitive hydrogenases.

##### 3.3.1.3. Expression of proteorhodopsin as alternative light activated bioenergetic machinery

A potential strategy towards enhancing light energy utilization is the expression of proteorhodopsin - retinal complexes (PRrC). PRrC acts as light-driven proton pumps with maximum absorption peaks between 490 and 520 nm, matching with the fraction of light normally reflected by photosynthetic chlorophylls (450–550 nm). To date, PRrC systems have been successfully expressed in *Synechocystis*PCC 6803 and obtained strains were able to stimulate photoheterotrophic growth in a PSI deficient strain. Nonetheless further growth improvements were halted by the kinetic limitations of the native NDH-1 complex for reversed NADP^+^ reduction. Surprisingly, although the provided additional PMF can increase the synthesis of ATP, thus enhancing LET via NADPH:ATP ratio balancing, during illumination with green light LET was reduced, mainly due to increased proton motive force (PMF) giving rise to cytochrome b6/f complex inhibition (Chen et al., 2017, 2019). This finding indicate that the integration of this systems should aim for a holistic comprehension of the overall bioenergetic context of the cells. In light of these findings, PRrC expression may expand the overall harvested light energy by filling the photosynthetic green gap with alternative light-driven proteins.

#### 3.3.2. Rewiring electron channeling

Solar energy is harvested by the photosynthetic apparatus and converted into reducing power. However, in contrast to the fixed carbon which will be converted into different metabolites and can be stored, reducing power in the form of electrons must be used upon generation. In order to keep generation and demand(s) balanced they are channeled to available electron sinks. In photosynthetic microorganisms the four main electron sinks are: Carbon fixation (the Calvin–Benson–Bassham cycle, CBB), nitrate reduction pathways, oxygen reduction pathways (respiration or photoprotective mechanisms) and hydrogen production (Kosourov et al., 2021). Furthermore, it has been demonstrated that preferential channeling of photosynthetic electrons towards native growth supporting pathways (mainly carbon fixation) hinders hydrogen production and promote hydrogen uptake even before the O_2_-driven hydrogenase deactivation takes place (Milrad et al., 2018). Therefore, rewiring the redox metabolism for a broader electron distribution towards hydrogen production is a crucial need for efficient photobiological H_2_ production. Multiple strategies exist to address these requirements.

##### 3.3.2.1. Inhibition of nitrogen metabolism

Nitrogen, specifically nitrate assimilation reactions account 16–20% of the photosynthetic electrons (Grund et al., 2019). Thus, inhibition of nitrate and nitrite reduction reactions has shown to translate into increased H_2_ production (Gutthann et al., 2007). A *Synechocystis* PCC 6803 mutant with the nitrate and nitrite reductase genes knocked-out showed an up to 140-fold increase in H_2_ production from the native bidirectional [NiFe] hydrogenase when compared to the WT cells. Interestingly, when cells where grown in media lacking nitrate, the engineered cells showed a 10-fold increase in H_2_ production compared to WT cells. Since the transcript levels for the native hydrogenase where comparable between WT and the mutant strain, the increased H_2_ production can be attributed to an increased availability of electrons (Baebprasert et al., 2011).

##### 3.3.2.2. Elimination of oxygen reduction pathways and photoprotective oxygen reduction

Photoprotective mechanisms allows photosynthetic cells to cope with sudden changes in light irradiation and subsequent variations in reducing power generation. However, they are also responsible for significant energy dissipation. In fact, most of these enzymes catalyze O_2_ reduction at the expense of H^+^ and electron consumption which could be otherwise redirected to H_2_ production. Several studies have reported the relevance of flavodiiron proteins (FDPs) (Santana-Sanchez et al., 2019) and terminal oxidases (Lea-Smith et al., 2013) as crucial mechanisms enabling cell survival in natural environments. Mutant strains lacking FDPs or terminal oxidases did not show significant growth defects when grown in controlled illumination conditions, highlighting their main role as electron-release valves under periods of reducing power overproduction.

Early studies in cyanobacteria showed improvements in H_2_ production in a *Synechocystis* PCC 6803 mutant lacking a functional NAD(P)H dehydrogenase complex NDH-1. Mutant strains sustained significant H_2_ production in the light. Since NDH-1 has been shown to be one of the major contributors to PMF formation by cyclic electron transport (CET) in *Synechocystis* PCC 6803 (Miller et al., 2021), the mutants are unable to pump protons, causing to severe alteration of PETC regulation and eventually increasing H_2_ production due to the long-term accumulation a highly reduced NADPH pool during illumination, being sufficient to activate NADPH-dependent H_2_ production by the native reversible hydrogenases (Cournac et al., 2004). Similarly, H_2_ production in *Synechocystis* PCC 6803 knock-out strains for terminal cytochrome c oxidase (Δ*cox*), terminal quinol oxidase (Δ*cyd*), and alternative respiratory terminal oxidase (Δ*ARTO*) all exhibited increased H_2_ production reaching up to 12-fold from WT levels for the triple mutant. Comparatively, the NDH-1 complex deficient mutant showed a 36-fold increase in total H_2_ evolution but the production rate was lower when compared to previous mutant strains (Gutthann et al., 2007). While the mechanism behind the enhanced H_2_ production in the terminal oxidase mutants seems to be mainly derived from enhanced electron supply to the hydrogenase, NDH-1 mutants may have other mechanisms. In the NDH1-mutants a lowered consumption of NADPH and reduced PMF derived from CET leads to an accumulation of NADPH which may be the main cause for enhanced production. Thus, it should be considered that altering the functioning of these respiratory photoprotective systems may not only increase H_2_ production, but also have a profound impact in overall redox balance and PETC regulation specially in more complex hosts as green algae (Elman et al., 2022). Other studies showed how deletion of FDPs in green algae lead to up more than 2-fold increase in H_2_ production under controlled pulse-illumination protocols. In addition, the lack of FDPs lead to a delayed activation of carbon fixation pathways (Jokel et al., 2019).

Considering these findings, deletion of respiratory and/or photoprotective electron sinks are powerful strategies to enhance H_2_ production by the increasing electron availability. Nonetheless, the impact of these deletions on cell fitness and robustness should being considered. We note that potential fitness-rescue effects may be observed when deletions are combined with introduced engineered electron sink such as a hydrogenase.

##### 3.3.2.3. Downregulation of carbon fixation pathways

Carbon fixation is essential for cell growth, where key reactions in the CBB cycle are energetically expensive consuming under ideal conditions 3 mol of ATP and 2 mol of NADPH per every mol of CO_2_ fixed. Consequently, it is not surprising that early studies on hydrogenase-catalyzed H_2_ production mainly focused on avoiding CBB activation via different physiological regulation mechanisms (Melis et al., 2000; Cournac et al., 2004). More recently, an RNA interference was used to downregulate expression of FNR in *Chlamydomonas reinhardtii*. Despite the small differences (12–18%) in autotrophic growth rates between WT and downregulated FNR mutants, a 60% reduction in Rubisco levels, 40% lower O_2_ evolution at PSII, and up to 140% higher starch degradation rates were observed which lead to an earlier onset of anaerobiosis and increased electron supply to the hydrogenases, translating into a 2.5-fold increase in overall H_2_ production (Sun et al., 2013). To our knowledge this is the only report of successful FNR regulation control in green algae via genetic engineering, leading to higher H_2_ production. In a similar fashion, a petH gene merodiploid *Synechocystis* PCC 6803 mutant, also exhibited up to 4-fold higher photobiological H_2_ production than the wild-type. In this study, the partial knock-out of the native FNR not only enhanced the H_2_ production but also lead to a longer duration of the transitory hydrogen production (Gutekunst et al., 2014). In this light, FNR downregulation is another promising strategy to increase photobiological hydrogen production.

#### 3.3.3. Engineering protective mechanisms against O_2_-inactivaton

As the ultimate aim of STH is using sunlight to source H_2_ from water, the process should mostly rely on LET where photosynthetic electrons are mostly sourced from water oxidation at PSII. However, increased PSII activity translates in higher O_2_ production, impairing H_2_ production due to the O_2_-sensitive nature of most hydrogenases (Vuorijoki et al., 2017; Kanygin et al., 2020, 2022). Albeit the relevance of dealing with O_2_ inactivation has been widely remarked, there are only a few studies reporting strategies tackling the O_2_-sensitivity issue by other means than expressing more O_2_-tolerant hydrogenases or anoxic cultivation conditions.

##### 3.3.3.1. Spatial confinement of hydrogenases in anoxic compartments

One possibility consists in expressing hydrogenases within an anoxic environment. In this regard, the heterocysts of filamentous nitrogen-fixing cyanobacteria could be used (Gärtner et al., 2012; Avilan et al., 2018). Although most described strategies showed limited H_2_ production, indicating that further engineering of the described systems is required. Similarly, expression of hydrogenases within anoxic bacterial microcompartments have already shown promising results in non-photosynthetic organisms. Particularly, carboxysomes are a very interesting scaffold for further engineering. Carboxysomes, present in all cyanobacteria, are polyhedral protein microcompartments that are selectively permeable to HCO_3_^−^ and H^+^ but not to O_2_ or CO_2_. Natively carboxysomes are formed by the self-assembly of different protein subunits, incorporating bicarbonate transporters, carbonic anhydrase and Rubisco (ribulose-1,5-bisphosphate carboxylase/oxygenase) within the compartment as a way to provide an anoxic environment and enhance carbon fixation. Interestingly, carboxysomes can be engineered to also incorporate heterologous enzymes within them. Due to the additional protection against O_2_ they are emerging as promising scaffolds for improving hydrogenase activity. α-carboxysome shells have been engineered to co-encapsulate a protein fusion composed of the [FeFe] hydrogenase HydA1 from *Chlamydomonas reinhardtii* and its native Fd together with FNR from *E. coli*. In this system, NADPH is used as soluble electron donor and FNR enables NADPH dependent electron transfer to the hydrogenase. Interestingly, this system exhibited increased H_2_ production under aerobic conditions both *in vivo* and *in vitro* (Li et al., 2020). Similarly, the catalytic subunits of the NADPH-dependent [NiFe] hydrogenase HyaAB from *E. coli* where expressed inside α-carboxysome shells. The assembled systems showed increased thermal stability and O_2_ tolerance both *in vivo* and *in vitro* when compared with the free enzyme causing a significant increase in the measured H_2_ production (Jiang et al., 2023). Nonetheless, this improvements in O_2_ tolerance were more significant for the [NiFe] hydrogenase HyaAB than the [FeFe] hydrogenase HydA1. These results indicate that even when protected in a carboxysome shell, the hydrogenase intrinsic O_2_ sensitivity still a critical factor in the catalytic system performance. Additionally, it should be noted that the dissolved O_2_ levels in the aerobic cultures were significantly lower than atmospheric, due to the respiratory metabolism of *E. coli*. Because of this, although highly promising, whether these strategies can be transferred to photosynthetic microorganisms and efficiently coupled to native redox metabolism remains to be verified.

##### 3.3.3.2. Implementation of O_2_ consuming pathways

An artificial O_2_ consumption pathway was integrated in *Chlamydomonas* chloroplasts, expressing *E. coli* pyruvate oxidase and *Synechococcus elongatus* PCC 7942 catalase under the control of a strong heat shock inducing promoter. The mutant strain exhibited higher oxygen consumption rate without significant growth defects or disruptions in the overall photosynthetic rate. The reduced intracellular O_2_ concentration increased hydrogen production at moderate light intensities (30 μE) by 2-fold factor compared to WT. However, at higher light intensities (100 μE) O_2_ production at PSII overcame the effects of the implemented O_2_ consumption pathway, indicating the need of further refinement of the system (Xu et al., 2011). Recently, efficient O_2_ consuming devices have been designed and characterized in *E. coli* (Pacheco et al., 2018). However, to our knowledge there are no reports about their implementation in any photosynthetic microorganism. In addition, respiration in photosynthetic organisms plays a key role in regulating intracellular O_2_ (Shimakawa et al., 2020) and could potentially also be considered a potential engineering target. Eventually, the expression of oxygen-binding proteins has also been proven to reduce hydrogenase inhibition. The heterologous expression and assembly of the oxygen binding protein leghemoglobin from *Glycine max*, in chloroplasts of *C. reinhardtii* decreased the intracellular O_2_ content and increased H_2_ production rate up to 1.5-fold WT levels (Wu et al., 2010). Similar effects were observed for the expression of cyanoglobin in heterocysts of *Nostoc* PCC 7120 (Avilan et al., 2018).

#### 3.3.4. Optimization of metal cofactors availability

Hydrogenases, their maturation machinery and electron carrier proteins all contain FeS clusters. Which is why it is expected that engineered strains overexpressing hydrogenases will feature a higher demand of FeS clusters. Therefore increasing native FeS cluster biosynthesis could potentially improve the system performance, as it has been already shown for nitrogenase enzymes (Li et al., 2016). In addition, as demonstrated by Akhtar and Jones (2008), deletion of *iscR* encoding the main FeS cluster biosynthesis pathway (ISC) repressor in *E. coli* increased hydrogenase activity and H_2_ accumulation. Besides the common heterotrophic pathways, photosynthetic organisms use the sulfur utilization factor (SUF) pathway as the main FeS cluster biosynthesis route and exhibit up to a 10-fold higher FeS cluster biosynthesis than *E. coli* (Gao, 2020). Similar studies in *Synechocystis* PCC 6803, showed that deletion of the SUF pathway repressor under sufficient iron conditions (SufR) lead to an increased FeS cluster assembly while maintaining overall cell fitness (Vuorijoki et al., 2017). In the light of these findings, deregulation of FeS cluster biosynthesis may be used as a promising strategy alleviate limitations in FeS cluster availability for expressed heterologous hydrogenases and the accessory proteins required for its functioning.

#### 3.3.5. Other engineering strategies

Future extensively engineered strains may exhibit limited growth, hindering the development of robust bioprocess design. This issue can be alleviated by implementing control systems for the expression of the introduced genetic elements. In this regard, the utilization of recently stationary phase promoters (Madsen et al., 2021) can drive the development of 2-stage cultivation strategies where initial growth is decoupled from further H_2_ production (Burg et al., 2016; Monshupanee et al., 2016). Additionally utilization of an O_2_-inducible promoter (Immethun et al., 2016) could be used for fine-control of O_2_ protective engineered systems.

## 4. Discussion

Future endeavors towards cost-effective photobiological H_2_ production must rely on a holistic approach combining the strategies highlighted in this review. Three main engineering directions have been identified. Namely, the expression of non-native hydrogenase with improved features, extensive modifications of PETC for focusing electrons towards the heterologous hydrogenase, and further physiological engineering strategies to improve the overall performance of the producing strains and the expressed hydrogenase.

Firstly, H_2_ productivity is highly dependent on the hydrogenase activity, requiring enzymes with displaced catalytic bias towards H_2_ production combined with O_2_ insensitivity. In this light [FeFe] hydrogenases are the most promising biocatalysts, for which recent research reported novel heterologous hydrogenases with intrinsic O_2_ tolerance as well as engineered fusion hydrogenase proteins leading to improved H_2_ production. Regarding fusion hydrogenases, it has been shown that protein design is critical. Any hydrogenase fusion protein must ensure efficient maturation of the hydrogenase H-domain and integration within the host bioenergetic machinery, ideally establishing unidirectional electron transport towards H_2_ production.

Secondly, efficient focusing of photosynthetic electrons towards the hydrogenase can be achieved via the implementation of the formerly described strategies. Nonetheless, implementing multiple engineering strategies could significantly impact the overall photosynthesis physiology, leading to, e.g., alterations in PSI to PSII ratios and consequently the balance between PMF, LET and CET. These effects are especially relevant for PSI-hydrogenase fusion proteins and knock-out strains of respiratory terminal oxidases and FDPs. Due to potential synergistic effects this should be considered.

Likewise, it must be considered the physiological context of the producing strain. In this regard further host engineering should be implemented to ensure maximum hydrogenase activity. Therefore, establishing an efficient and orthogonal maturation systems is evidently required, especially in the case of [FeFe] hydrogenases expression in cyanobacteria. Moreover, O_2_ protection strategies should be implemented to avoid irreversible hydrogenase inactivation, as this is likely to remain an issue even in designed enzymes.

Eventually, it is crucial to distinguish between H_2_ production derived from the LET and other sources. Albeit many studies reported that reducing PSII activity correlated with higher H_2_ production due to reduced intracellular O_2_ concentrations this derives from overall lower water splitting at PSII and thus reduced LET, which ultimately limits the maximum theoretical STH H_2_ production. This long-sustained paradox between LET activity and H_2_ production may be overcome by the implementation of O_2_ consuming device, pathway or the assembly of an O_2_ protection mechanism *in vivo* thereby avoiding hydrogenase inactivation and efficiently coupling water splitting with H_2_ production.

Finally, future efforts to improve photobiological H_2_ production should use a combination of strategies highlighted in the study. These strategies include expressing and efficiently activating heterologous hydrogenases, avoiding its O_2_-inactivation and levering the limitations of PETC towards increasing electron channeling to the hydrogenase. The described strategies can significantly impact overall photosynthesis physiology. Namely alterations in PSI to PSII ratios and balance between PMF, LET and CET. Thus, the interaction between the different engineering strategies must consider its synergistic effects towards the development of cost-effective photobiological H_2_ production processes.

## Author contributions

All authors contributed to conception and design of the review. CS and JF wrote the first draft of the manuscript. GB and PL contributed to read, revision and proof-reading of the manuscript drafts. CS contributed to the literature sources collection regarding hydrogenases. JF contributed to the literature sources collection of photosynthesis re-engineering and heterologous expression of hydrogenases. All authors contributed to the article and approved the submitted version.

## Funding

This work was supported by the European Union Horizon Europe - the Framework Programme for Research and Innovation (2021–2027) under the grant agreement number 101070948 (project PhotoSynH2 to GB and PL) and The Swedish Energy Agency (project number 48574-1 to GB).

## Conflict of interest

The authors declare that the research was conducted in the absence of any commercial or financial relationships that could be construed as a potential conflict of interest.

## Publisher’s note

All claims expressed in this article are solely those of the authors and do not necessarily represent those of their affiliated organizations, or those of the publisher, the editors and the reviewers. Any product that may be evaluated in this article, or claim that may be made by its manufacturer, is not guaranteed or endorsed by the publisher.
